# Supplementary material for: Modeling glioblastoma heterogeneity as a dynamic network of cell states
Source: Mol Syst Biol. 2021 Sep 16;17(9):e10105. doi: 10.15252/msb.202010105 (PMC8444284; doi:10.15252/msb.202010105)
Supplement: Supplementary file 6 — Source Data for Figure 5 [file MSB-17-e10105-s004.zip › Figure5A_sourcedata/GSEA_3071/hallmarks_stateIII.GseaPreranked.1623742540963/HALLMARK_APOPTOSIS.html]

Details for gene set HALLMARK\_APOPTOSIS[GSEA]

|  || Dataset | state33071 |
| Phenotype | NoPhenotypeAvailable |
| Upregulated in class | na\_neg |
| GeneSet | HALLMARK\_APOPTOSIS |
| Enrichment Score (ES) | -0.33136213 |
| Normalized Enrichment Score (NES) | -1.8025758 |
| Nominal p-value | 0.0021413276 |
| FDR q-value | 0.025217699 |
| FWER p-Value | 0.282 |
Table: GSEA Results Summary

  

Fig 1: Enrichment plot: HALLMARK\_APOPTOSIS      
 Profile of the Running ES Score & Positions of GeneSet Members on the Rank Ordered List

  

| PROBE | GENE SYMBOL | GENE\_TITLE | RANK IN GENE LIST | RANK METRIC SCORE | RUNNING ES | CORE ENRICHMENT || 1 | HSPB1 |  |  | 4 | 0.650 | 0.0260 | No |
| 2 | DAP |  |  | 193 | 0.342 | -0.0083 | No |
| 3 | SOD1 |  |  | 315 | 0.287 | -0.0275 | No |
| 4 | TSPO |  |  | 341 | 0.280 | -0.0223 | No |
| 5 | DNAJA1 |  |  | 355 | 0.273 | -0.0143 | No |
| 6 | PPP2R5B |  |  | 489 | -0.254 | -0.0381 | No |
| 7 | RNASEL |  |  | 505 | -0.255 | -0.0314 | No |
| 8 | ATF3 |  |  | 515 | -0.255 | -0.0231 | No |
| 9 | BCAP31 |  |  | 542 | -0.256 | -0.0191 | No |
| 10 | SC5D |  |  | 756 | -0.267 | -0.0630 | No |
| 11 | DNAJC3 |  |  | 780 | -0.268 | -0.0577 | No |
| 12 | CASP8 |  |  | 820 | -0.270 | -0.0566 | No |
| 13 | RHOB |  |  | 865 | -0.272 | -0.0566 | No |
| 14 | FEZ1 |  |  | 1005 | -0.280 | -0.0808 | No |
| 15 | CDC25B |  |  | 1251 | -0.292 | -0.1319 | No |
| 16 | BTG2 |  |  | 1350 | -0.297 | -0.1448 | No |
| 17 | PSEN2 |  |  | 1421 | -0.301 | -0.1503 | No |
| 18 | IGF2R |  |  | 1432 | -0.302 | -0.1403 | No |
| 19 | BIRC3 |  |  | 1469 | -0.304 | -0.1370 | No |
| 20 | BCL2L11 |  |  | 1542 | -0.308 | -0.1427 | No |
| 21 | CDKN1A |  |  | 1631 | -0.314 | -0.1523 | No |
| 22 | GPX3 |  |  | 1638 | -0.315 | -0.1408 | No |
| 23 | BRCA1 |  |  | 1844 | -0.327 | -0.1801 | No |
| 24 | ANKH |  |  | 1978 | -0.334 | -0.2005 | No |
| 25 | TIMP1 |  |  | 2038 | -0.337 | -0.2017 | No |
| 26 | ENO2 |  |  | 2366 | -0.363 | -0.2710 | No |
| 27 | HMOX1 |  |  | 2415 | -0.366 | -0.2682 | No |
| 28 | CD44 |  |  | 2471 | -0.371 | -0.2669 | No |
| 29 | BCL2L2 |  |  | 2522 | -0.375 | -0.2642 | No |
| 30 | MMP2 |  |  | 2527 | -0.375 | -0.2496 | No |
| 31 | ERBB2 |  |  | 2680 | -0.389 | -0.2727 | No |
| 32 | MADD |  |  | 2783 | -0.397 | -0.2825 | No |
| 33 | CYLD |  |  | 2838 | -0.402 | -0.2797 | No |
| 34 | JUN |  |  | 2935 | -0.413 | -0.2873 | No |
| 35 | EMP1 |  |  | 2998 | -0.420 | -0.2858 | No |
| 36 | CCND2 |  |  | 3037 | -0.425 | -0.2779 | No |
| 37 | RETSAT |  |  | 3245 | -0.450 | -0.3126 | Yes |
| 38 | BMP2 |  |  | 3307 | -0.462 | -0.3091 | Yes |
| 39 | SLC20A1 |  |  | 3344 | -0.470 | -0.2989 | Yes |
| 40 | CTH |  |  | 3386 | -0.478 | -0.2896 | Yes |
| 41 | TNFSF10 |  |  | 3485 | -0.502 | -0.2939 | Yes |
| 42 | F2R |  |  | 3538 | -0.523 | -0.2856 | Yes |
| 43 | TGFBR3 |  |  | 3546 | -0.525 | -0.2655 | Yes |
| 44 | PLAT |  |  | 3600 | -0.547 | -0.2564 | Yes |
| 45 | ERBB3 |  |  | 3617 | -0.558 | -0.2373 | Yes |
| 46 | HGF |  |  | 3662 | -0.585 | -0.2243 | Yes |
| 47 | TOP2A |  |  | 3671 | -0.594 | -0.2016 | Yes |
| 48 | PDGFRB |  |  | 3678 | -0.598 | -0.1783 | Yes |
| 49 | DCN |  |  | 3723 | -0.633 | -0.1633 | Yes |
| 50 | SATB1 |  |  | 3732 | -0.644 | -0.1385 | Yes |
| 51 | TGFB2 |  |  | 3746 | -0.667 | -0.1141 | Yes |
| 52 | TIMP3 |  |  | 3755 | -0.677 | -0.0880 | Yes |
| 53 | APP |  |  | 3779 | -0.710 | -0.0644 | Yes |
| 54 | BGN |  |  | 3812 | -0.761 | -0.0409 | Yes |
| 55 | CLU |  |  | 3846 | -0.827 | -0.0150 | Yes |
| 56 | LUM |  |  | 3861 | -0.870 | 0.0176 | Yes |
Table: GSEA details [plain text format]

  

Fig 2: HALLMARK\_APOPTOSIS: Random ES distribution      
 Gene set null distribution of ES for **HALLMARK\_APOPTOSIS**
